# Effect of Green Tea Kombucha Within an Energy‐Restricted Diet on Cardiometabolic Risk Markers in Individuals With Excess Body Weight: A Randomized Controlled Trial

**DOI:** 10.1111/1750-3841.71050

**Published:** 2026-04-09

**Authors:** Dandara Baia Bonifácio, Gabriela Macedo Fraiz, Udielle Vermelho Lacerda, Rodrigo Rezende Cardoso, Thayná de Souza Coura Dias, Viviana Corich, Alessio Giacomini, Frederico Augusto Ribeiro de Barros, Josefina Bressan

**Affiliations:** ^1^ Department of Nutrition and Health Universidade Federal de Viçosa Viçosa Brazil; ^2^ Department of Nutrition, Food Science and Physiology, Centre for Nutrition Research University of Navarra Pamplona Spain; ^3^ Department of Food and Technology Universidade Federal de Viçosa Viçosa Brazil; ^4^ Department of Agronomy, Food Natural Resources, and Environment (DAFNAE) Università degli Studi di Padova Legnaro Italy

**Keywords:** cardiovascular diseases, glycated hemoglobin, lipids, uric acid

## Abstract

Green tea kombucha is a fermented beverage notable for its rich content of bioactive compounds, yet it remains underexplored. Thus, this study aimed to evaluate the effect of green tea kombucha consumption, in combination with an energy‐restricted diet, on traditional cardiometabolic risk markers in individuals with excess weight. This is a randomized controlled clinical trial in which 59 individuals with excess body weight (BMI >27 kg/m^2^) were distributed into two parallel experimental groups: the control group, which followed an energy‐restricted diet (−500 kcal/day), and the kombucha group, which followed the same energy‐restricted diet + daily consumption of green tea kombucha (200 mL/day) for 10 weeks. At the end of the study, participants in the kombucha group showed a significant reduction in total cholesterol (*p* = 0.024), LDL‐c (*p* = 0.035), VLDL‐c (*p* = 0.011), triglycerides (*p* = 0.012), Castelli index II (*p* = 0.002), and uric acid (*p* = 0.027) compared to baseline. Both groups (control and kombucha) significantly reduced Castelli index I (*p* = 0.015 and *p* = 0.007, respectively) and HOMA‐β (*p* = 0.019 and *p* < 0.001, respectively) after the intervention. Only the control group showed a significant increase in glycated hemoglobin (within reference values) compared to pre‐intervention (*p* = 0.004). Furthermore, men in the kombucha group showed a significant reduction in glycated hemoglobin compared to men in the control group (*p* = 0.010). However, in the total sample (*Δ* = final − initial), no significant differences were found between the control and kombucha groups (*p* > 0.05) for any evaluated marker. In conclusion, the consumption of green tea kombucha, when combined with an energy‐restricted diet, shows promising cardiometabolic benefits in individuals with excess body weight.

## 1 Introduction

Excess body weight is defined as a chronic and multifactorial condition associated with the development of morbidities associated with the accumulation of body fat (Tchang et al. 2021). It reaches endemic proportions in many parts of the world. In the European region, 60% of adults have excess weight, which has been one of the main determinants of disability and death (The Lancet Public Health 2023). The accumulation of body fat is characterized as a risk factor for the development of cardiovascular diseases through the possibility of the coexistence of insulin resistance, hyperglycemia, hypertension, and dyslipidemia (Bovolini et al. 2021). Furthermore, excess body weight can cause structural and functional changes to accommodate body weight that impair vascular homeostasis (Koliaki et al. 2019). For these reasons, people with excess body weight have a higher risk of death from cardiovascular diseases, independent of other metabolic factors (Powell‐Wiley et al. 2021). In this sense, the treatment of excess body weight, with an energy‐restricted diet, is necessary to reduce the risk of developing cardiovascular diseases, with atherosclerosis being one of the most significant (Asgary et al. 2018).

To minimize the impacts of excess body weight, studies have focused on evaluating the effects of specific foods on cardiometabolic risk markers, particularly by improving lipid and glucose profiles (Sikand et al. 2015; Asgary et al. 2018). In this context, kombucha stands out among fermented foods rich in phenolic compounds. This beverage is produced by fermentation of *Camellia sinensis* tea (mainly green and black tea) with sugars using a symbiotic culture of bacteria and yeast known as SCOBY (Cardoso et al. 2020). In its rich composition, phenolic compounds (e.g. catechins) and organic acids such as acetic, citric, gluconic, carbonic, tartaric, and lactic acids may play a key role in the beverage's therapeutic action (Júnior et al. 2022). Thus, the rich composition of kombucha may be responsible for the promising perspectives on its effect on human health.

Two recent clinical studies highlighted the hypoglycemic potential of kombucha (Atkinson et al. 2023; Mendelson et al. 2023). However, there are still few studies that evaluated the effect of kombucha consumption on cardiometabolic human health. Thus, the objective of the present study was to evaluate the potential additional effect of daily consumption of 200 mL of green tea kombucha, alongside of an isocaloric energy‐restricted diet, for 10 weeks, on cardiometabolic markers in individuals with excess weight. The strategy of adopting an energy‐restricted diet in both groups is because it is a commonly consolidated strategy for reducing cardiometabolic risk markers in overweight individuals, without apparent comorbidities.

The hypothesis of this study is that the rich composition of green tea kombucha may help in the improvement of cardiometabolic risk markers in individuals with excess body weight. By assessing the potential effect of green tea kombucha, this study will help to fill the gap in the literature on the impact of fermented beverages and dietary strategies in reducing the risk of comorbidities associated with overweight and obesity.

## 2 Materials and Methods

This is an open‐label randomized controlled clinical trial, with two parallel experimental groups, carried out with adults with excess body weight for 10 weeks. This work was carried out at the Federal University of Viçosa (Minas Gerais, Brazil), through partnerships between the Department of Nutrition and Health and the Department of Food Technology. This study is a substudy of a broader investigation on the effect of green tea kombucha consumption on weight loss. Further methodological details are available in Fraiz et al. (2024).

This study was approved by the National Research Ethics Commission (CONEP) (CAAE: 25880819.3.0000.5153; number: 3.948.033), by the Brazilian Clinical Trials Registry (REBEC) (number: RBR‐9832wsx), and is in accordance with the Resolution CNS/466 of 2012 and Declaration of Helsinki. All participants who agreed to participate in the study were informed about the risks and benefits and signed an informed consent form.

### 2.1 Participants

This study was conducted with adult individuals, aged between 18 and 45 years, who had a body mass index (BMI) ≥27 kg/m^2^, body fat percentage >25% for men and >30% for women, waist circumference ≥94 cm for men and ≥80 cm for women and no chronic or acute illness in the last 3 months. Pregnant, lactating, or menopausal women, smokers, and those using medications such as antibiotics, anti‐inflammatories, corticosteroids, nutritional supplements, teas, kombucha, drugs, and alcohol abuse were not included. Furthermore, people with eating disorders, vegans, and athletes were not included.

### 2.2 Sample Calculation and Randomization

The sample size calculation was based on the primary outcome, Δ body fat values, as previously reported in published results from this RCT (12). Briefly, the calculation was performed using G*Power software (version 3.1.9.6), assuming an effect size of 0.7, a power of 80%, and a significance level of 5%. This resulted in a required sample size of 31 individuals per group (Fraiz et al. 2024).

Participants were randomized in a 1:1 ratio, using the minimization method in the MinimPy software, version 0.2 (Copyright, Mahmoud Saghaei, 2010–2011), considering sex, age, and BMI as prognostic factors (Abramson 2011). Then, according to randomization, participants were allocated into two parallel groups—the control group, which followed an energy‐restricted diet (−500 kcal/day), or the kombucha group, which in addition to calorie restriction (−500 kcal/day), received 200 mL of green tea kombucha daily, for 10 weeks.

### 2.3 Study Design

Participant recruitment took place in the local community, and those who showed interest completed an online pre‐screening questionnaire. Those who met the initial inclusion criteria attended the laboratory to confirm the information provided through body composition and anthropometric measurements, and also filled out the three‐factor eating questionnaire (TFEQ). Only those who obtained TFEQ scores that did not reveal possible eating disorders participated in the study. Eligible candidates who agreed to participate in the study went through the run‐in period to investigate and exclude those who presented the possibility of non‐adherence to the study protocol. During this period, participants had to maintain their usual diet and body weight without significant changes (<2%) for 1 week.

Participants answered the food frequency questionnaire (FFQ) and the International Physical Activity Questionnaire short version (IPAQ) in addition to blood collection. During 10 weeks, participants followed the interventions determined by the study and were recommended to maintain their usual level of physical activity. Halfway through the study, a follow‐up visit was conducted to assess weight, anthropometric measurements, and body composition, as well as to administer a questionnaire on adherence to the study protocol. Additionally, participants in the kombucha group received record sheets to track their kombucha consumption. Throughout the study, participants who were unable to adhere to the protocol, had any illness, or had any adverse reaction to kombucha consumption were discontinued from the study.

### 2.4 Diet Plan

The eating plans were prepared for each participant individually, considering the caloric needs determined by the estimated energy requirement (EER) for adults with excess weight, and the distribution guidelines for macronutrients and micronutrients necessary for maintaining health (Trumbo et al. 2002). For each participant, a calorie restriction of 500 kcal/day was applied. Energy‐restricted was applied to participants, as it is the gold‐standard intervention for improving metabolic parameters and promoting weight loss in individuals with excess weight. During the study, participants were instructed to avoid regular consumption of potentially functional foods, including nuts, high‐fiber flours, fish oils, and olive oil. All material offered, as well as menu calculations, was carried out by nutritionists.

### 2.5 Kombucha Production

**FIGURE 1:**
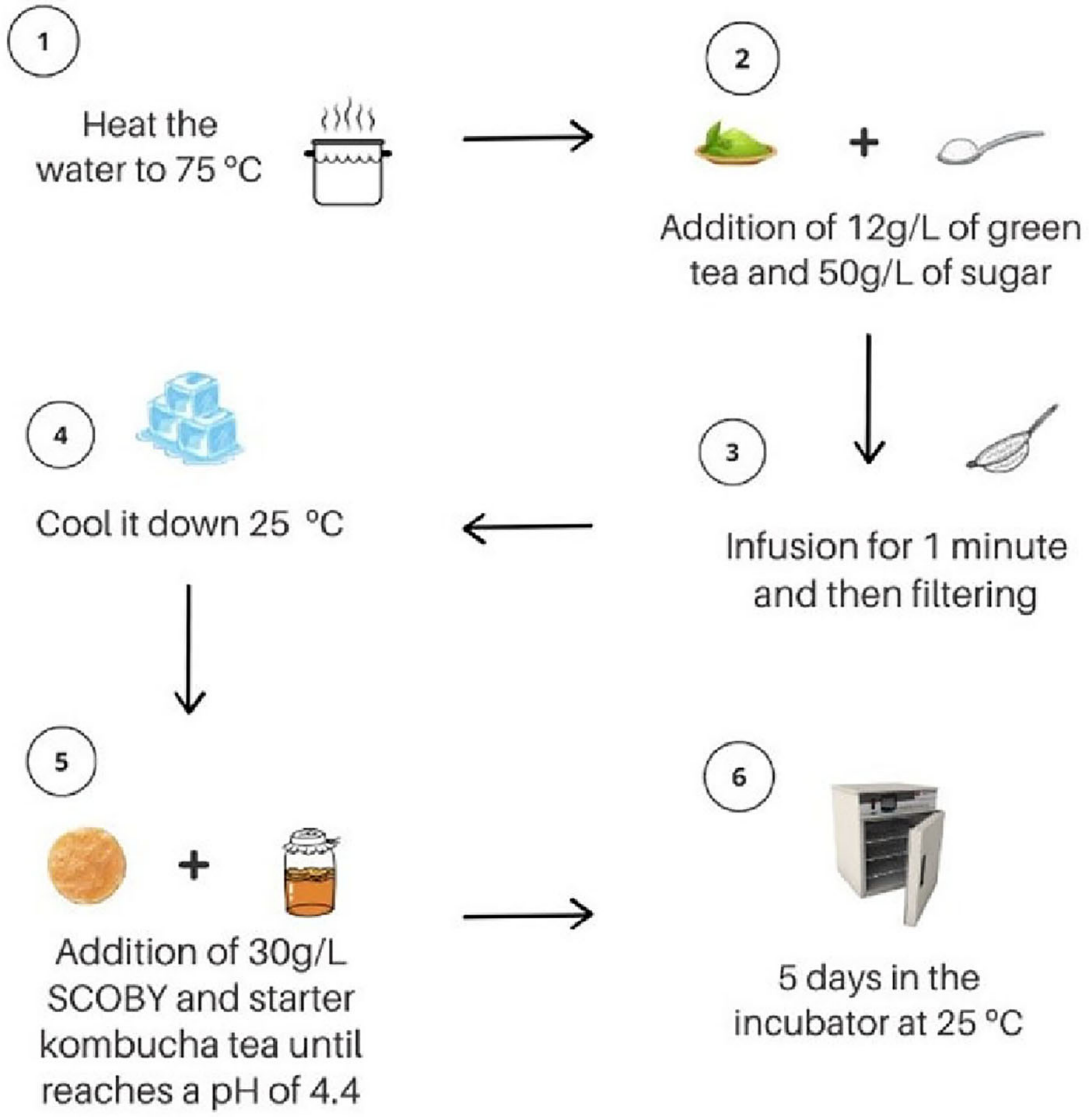
Procedures for producing green tea kombucha. °C: degrees Celsius; %: percentage; g/L: grams per liter; v/v: volume per volume.

The kombucha was prepared with Amaya brand green tea, grown in the city of Registro (São Paulo, Brazil). To prepare the tea infusion, initially, the water was heated to 75°C, then 12 g of green tea and 50 g of sugar were added per liter of water. The tea was filtered and immediately cooled to 25°C in an ice bath. Then, 30 g/L of SCOBY was added, as well as a previously produced kombucha, until it reached a pH of 4.4. Finally, the kombucha was fermented for 5 days at 25°C, portioned into 200 mL sterile plastic packaging, and distributed weekly to participants in the kombucha group, who were instructed to keep the beverage refrigerated (Figure 1). Furthermore, participants were advised to consume the beverage during the main meal.

At the end of preparation, the green tea kombucha had a pH of 3.41 ± 0.09 and a total acidity of 0.20 ± 0.02 (w/v acetic acid). Furthermore, the beverage had an antioxidant capacity of 3.24 ± 0.43 µmol TE/mL and a total phenolic content of 0.32 ± 0.003 mg/mL in gallic acid equivalents (GAE). Through microbiological characterization, populations of lactic acid bacteria (1.98 × 10^7^ CFU/mL), acetic bacteria (1.07 × 10^7^ CFU/mL), and yeast (1.57 × 10^7^ CFU/mL) were evaluated. Furthermore, 92 phenolic compounds were identified in the beverage through Ultra‐high performance liquid chromatography‐MS/MS (UPLC‐MS/MS) analysis, and among the main ones were gallocatechin, catechin 5‐O‐gallate, epicatechin, epigallocatechin, 5‐O‐galloylquinic acid, catechin, 5‐p‐coumaroylquinic acid, quercetin 3‐O‐rutinoside, and niricetin 3‐O‐glycoside. In total, 70.7% of these belonged to the flavonoid class, followed by phenolic acids (25%), lignans (2.2%), and other polyphenols (1.1%).

Details on the production process of green tea kombucha, as well as its biochemical and microbiological evaluations, are described in Fraiz et al. (2024).

### 2.6 Variables and Measurement Instruments

To evaluate the participants' characterization variables, during the baseline and run‐in period, weight and body composition were measured using the tetrapolar electrical bioimpedance technique (Tetrapolar Bioimpedance, Inbody, model Y230). Furthermore, with an inelastic measuring tape, the circumference of the waist, hips, and neck was measured with three repetitions. From the values obtained, the waist/hip ratio was calculated (Ministry of Health 2011).

The participants' food consumption was assessed using FFQ, which was digitized in ERICA‐REC24h, which uses the food composition table developed by the Brazilian Institute of Geography and Statistics (IBGE). Physical activity was assessed using the short version of the IPAQ, validated for the Brazilian population, and presented in the metabolic equivalent task (MET).

The last meal of the day before collection was a standardized dinner for all participants. Blood was collected by a qualified professional after a 10‐h overnight fast. After collection, the samples were analyzed at the Clinical Analysis Laboratory (LACDSA/UFV), following the laboratory's internal recommendations, and the results of tests for total cholesterol (TC), LDL‐c, high‐density lipoprotein (HDL‐c), very low‐density lipoprotein (VLDL‐c), triglycerides (TG), apolipoprotein A (Apo A), apolipoprotein B (Apo B), fasting blood glucose, estimated blood glucose, insulin, glycated hemoglobin and uric acid (UA) were sent to researchers. All markers mentioned were analyzed using the UV kinetic method with the BS 200 biochemical analyzing equipment. With the results of the aforementioned exams, it was possible to calculate the Castelli I and II indices, using the divisions of TC/HDL‐c and LDL‐c/HDL‐c respectively (Catelli 1988). The HOMA indices were also calculated using the following formulas: HOMA‐IR = [(fasting insulin (mU/L) × fasting blood glucose (mmol/L)/22.5] and HOMA‐β = [20 × fasting insulin (MICRO IU/mL)) / (fasting glucose (mmol/L*) − 3.5] (Matthews et al. 1985). Additionally, the TyG index (Simental‐Mendía et al. 2008) was also calculated according to the formula: TyG = Ln [(fasting triglycerides (mg/dL)) × (fasting blood glucose (mg/dL))/2].

### 2.7 Statistical Analyses

The statistical analyses of this study were carried out using SPSS software (version 25.0, USA), considering a statistical significance of 5%. Data normality was assessed using the Shapiro–Wilk test, and homoscedasticity using Levene's test. Paired *t*‐test and Wilcoxon test were used to evaluate the effect of time on treatments, while independent samples *t*‐test and Mann–Whitney U test were performed for comparisons between groups. Multifactorial ANOVA was conducted to evaluate the interaction between treatment and participants’ sex. When a significant interaction was observed, differences between the groups by sex were assessed using Bonferroni post‐hoc tests. The data from this study are presented as mean and standard deviation or median and minimum and maximum values.

## 3 Results

In total, 500 individuals completed the pre‐selection questionnaire, and only 75 met the inclusion criteria. A total of 37 individuals were randomized in the control group, and 38 to the kombucha group. In the end, 59 participants completed the intervention, 29 in the control group and 30 in the kombucha group. The CONSORT flow diagram of participants can be found in Fraiz et al. (2024). Among the causes of loss to follow‐up were personal reasons (43.75%), use of antibiotics (18.75%), anxiety crisis (6.25%), non‐adherence to the diet (6.25%), did not like the taste of kombucha (6.25%), COVID‐19 infection (6.25%), pregnancy (6.25%), and discontinuation of kombucha consumption (6.25%). Baseline values of all markers studied did not differ between those who started the study and those who completed the study (data not shown).

At the end of the study, the control group was composed of 17 women and 12 men, while the kombucha group presented 18 women and 12 men. Age, anthropometric, and body composition variables did not differ between participants at the beginning of the study (Table 1). After 10 weeks of intervention, both groups lost weight in a similar manner, as highlighted in Fraiz et al. (2024).

**TABLE 1:** Anthropometric and body composition characteristics of the control and kombucha groups at baseline.

Variables	Control group	Kombucha group	*P*
Age (years)	32.51 (6.9)	34.66 (6.9)	0.239
BMI (kg/m^2^)	32.05 (27.3–46.5)	32.90 (27.3–40.8)	0.259
Muscle mass (kg)	30.95 (21.2–44.4)	29.60 (22.3–52.7)	0.666
Body fat (%)	38.14 (8.2)	39.93 (6.7)	0.267
Neck circumference (cm)	36 (26.5–43.4)	36.5 (32.5–44)	0.804
Waist circumference (cm)	92.52 (8.5)	97.05 (10.7)	0.078
Hip circumference (cm)	114.75 (8.3)	115.47 (7.96)	0.816
Waist/hip ratio	0.89 (0.06)	0.92 (0.07)	0.451

Values are presented as mean (standard deviation) or median (minimum–maximum value). The *p* values represent the results obtained by the independent samples t‐test or Mann–Whitney U test. %—percentage; cm: centimeter; BMI: body mass index; kg: kilogram; kg/m^2^: kilogram per square meter.

Using the IPAQ, it was possible to verify that there was no difference between the initial and final level of physical activity, both in the control (*p* = 0.352) and in the kombucha group (*p* = 0.107). Furthermore, throughout the study, the groups did not differ in terms of food consumption and level of physical activity, measured by the FFQ and IPAQ, respectively (Table 2).

**TABLE 2:** Food consumption and physical activity level of participants (self‐reported) during the study according to allocation group.

Variable	Control group	Kombucha group	*p*
Calories (kcal)	1359 (399.4)	1432 (453.7)	0.736
Carbohydrate (g)	117.9 (47.5–319)	120.3 (56.4–310)	0.458
Protein (g)	44 (12.9–95.5)	38.4 (26.2–120)	0.891
Lipids (g)	36.57 (13.6–75.3)	32 (15.8–94.4)	0.585
Fibers (g)	12.29 (4.6–34.5)	13.09 (5.2–35.1)	0.275
PA—Walk (MET)	387 (655)	336 (545)	0.525
PA—Moderate (MET)	1360 (2620)	798 (901)	0.981
PA—Vigorous (MET)	4481 (8681)	4635 (8981)	0.524
PA—Total (MET)	6230 (9337)	5770 (9287)	0.649

Values are presented as mean (standard deviation) or median (minimum–maximum value). The *p* values represent the results obtained by the independent samples *t*‐test or Mann–Whitney U test. Food consumption results according to the food frequency questionnaire (FFQ), adjusted per thousand kilocalories. Physical activity results according to metabolic equivalent task (MET) scores obtained from the International Physical Activity Questionnaire (IPAQ). g: gram; Kcal: kilocalorie; PA: physical activity.

After intervention, in an intragroup analysis, it was possible to observe that the kombucha group reduced several markers of the lipid profile, such as TC, LDL‐c, VLDL‐c, TG, and Castelli index I and II, compared to baseline, while the control group reduced only the Castelli index I. Additionally, only the kombucha group reduced uric acid after intervention. Regarding the glucose metabolism, both groups reduced HOMA‐β, and only the control group increased glycated hemoglobin (within normal values) after 10 weeks. The other markers remained unchanged after the intervention.

**FIGURE 2:**
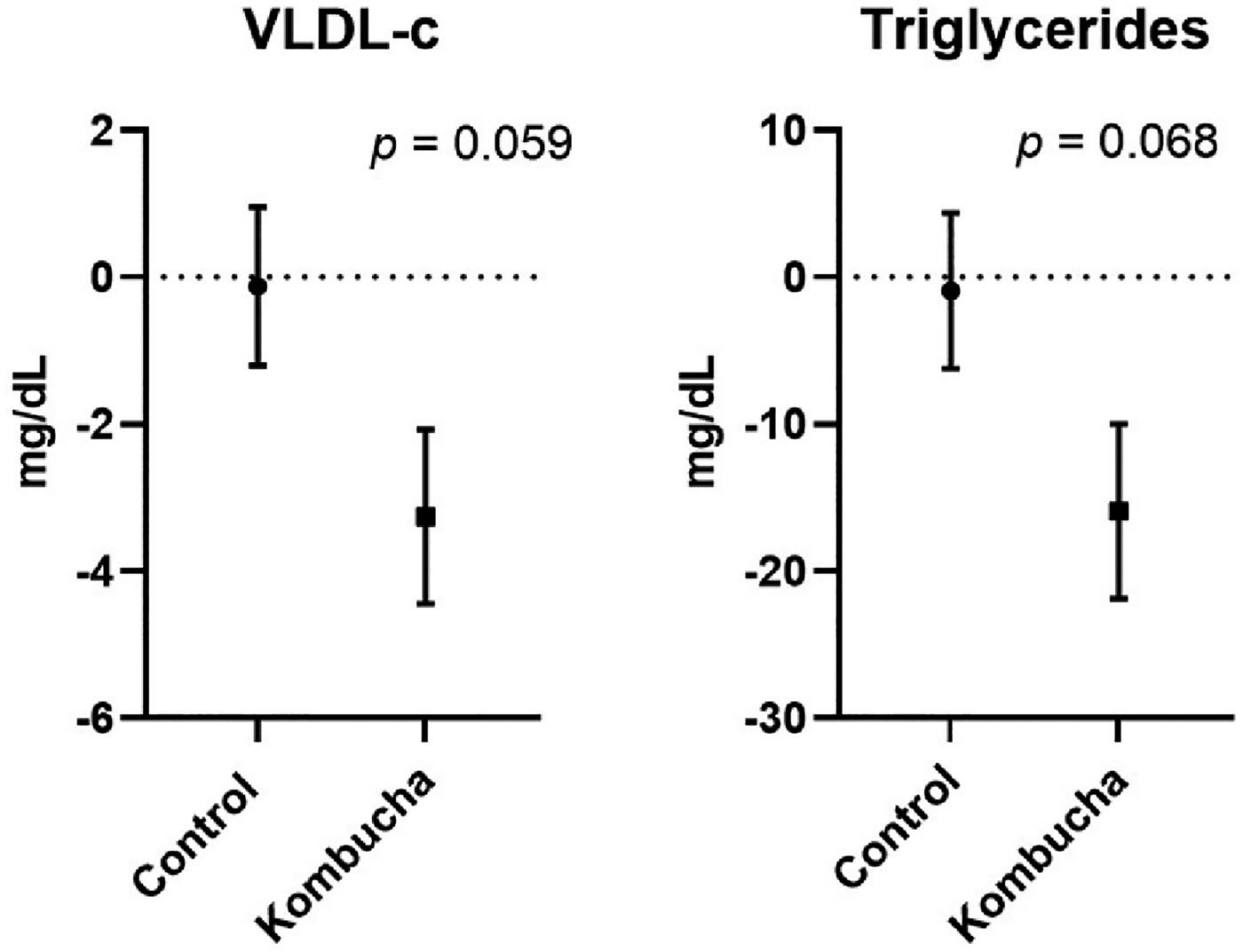
Impact of interventions on lipid profile markers showing a tendency to decrease after kombucha consumption. The p value represents the differences between the *∆* (final − initial) of the kombucha and control group in the independent samples *t*‐test. mg/dL: milligram per deciliter; VLDL‐c: very low‐density lipoprotein.

When comparing the differences between the groups in two moments (*∆* = final − initial), there were no differences between the control and kombucha for all evaluated variables (Table 3). However, a clinically greater reduction in VLDL‐c (−3.25 vs. −0.12 mg/dL; *p* = 0.059) and TG (−15.92 vs. −0.91 mg/dL; *p* = 0.068) was notable in participants in the kombucha group, compared to those in the control group. The variability of the data or the sample size likely influenced the lack of statistical significance (*p* < 0.05) for these variables (Figure 2).

**TABLE 3:** Effect of 10 weeks of intervention on cardiometabolic risk markers, according to allocation group.

Variable	Control initial	Control final	*p*‐value	Kombucha initial	Kombucha final	*p*‐value	∆ control	∆ kombucha	∆*p*‐value
TC (mg/dL)	180.60 (28.46)	177.57 (24.89)	0.352	182.92 (32.70)	173.53 (27.18)	**0.024**	−3.03 (16.94)	−9.39 (20.74)	0.215
LDL‐c (mg/dL)	113.39 (28.42)	110.32 (25.49)	0.290	114.48 (22.71)	108.36 (17.91)	**0.035**	−3.07 (15.05)	−6.12 (13.67)	0.446
HDL‐c (mg/dL)	39.56 (7.81)	41.24 (7.41)	0.093	40.79 (8.32)	42.31 (9.32)	0.250	1.68 (4.80)	1.51 (6.95)	0.922
VLDL‐c (mg/dL)	18.50 (10–43)	17.00 (10–54)	0.866	24.07 (10.39)	20.81 (7.69)	**0.011**	−0.12 (5.28)	−3.25 (6.16)	0.059
TG (mg/dL)	91.50 (52–216)	84.00 (49–269)	0.954	120.29 (52.02)	104.37 (38.40)	**0.012**	−0.91 (26.04)	−15.92 (30.82)	0.068
Castelli index I	4.62 (1.04)	4.38 (0.88)	**0.015**	4.66 (1.11)	4.29 (1.04)	**0.007**	−0.23 (0.44)	−0.36 (0.64)	0.423
Castelli index II	2.84 (0.72)	2.75 (0.73)	0.097	3.02 (0.75)	2.73 (0.73)	**0.002**	−0.09 (0.26)	−0.29 (0.38)	0.066
Apo A (mg/dL)	121 (108–208)	122 (101–239)	0.966	130.13 (20.22)	129.37 (18.97)	0.554	0.97 (12.27)	−0.77 (14.66)	0.728
Apo B (mg/dL)	97.96 (19.54)	100.48 (17.71)	0.332	100.48 (20.85)	99.92 (14.79)	0.838	0.00 (−20–33)	3.00 (–33–16)	0.869
Apo A/Apo B index	1.26 (0.82–1.81)	1.18 (0.88–1.86)	0.159	1.27 (0.30)	1.26 (0.21)	0.653	−0.06 (0.22)	−0.01 (0.16)	0.433
Fasting blood glucose (mg/dL)	80.8 (5.63)	86.6 (10.93)	0.052	86 (5.96)	91.81 (6.69)	0.057	5.8 (2.59)	5.8 (2.66)	1.000
Insulin (µUI/mL)	9.40 (3.39)	8.50 (3.03)	0.187	12.40 (6.10–19.20)	10.70 (4.50–24.80)	0.424	−0.90 (3.37)	−0.47 (4.07)	0.851
Glycated hemoglobin (%)	5.07 (0.29)	5.16 (0.29)	**0.004**	5.05 (0.32)	5.08 (0.33)	0.478	0.10 (−0.30–0.40)	0.05 (−0.30–0.30)	0.126
HOMA‐ IR	1.89 (0.70)	1.89 (0.73)	0.987	2.69 (1.14–4.50)	2.27 (1.33–5.93)	0.572	0.00 (0.71)	0.11 (1.02)	0.650
HOMA‐β	138.25 (56.04–293.05)	99.09 (45.77–267.20)	**0.019**	192.90 (95.84–357.75)	117.15 (59.59–263.34)	**<0.001**	−39.96 (66.59)	−66.43 (50.39)	0.134
Tyg	4.45 (4.21–4.91)	4.48 (4.17–4.99)	0.189	4.57 (0.23)	4.57 (0.20)	0.896	0.02 (0.09)	−0.00 (0.13)	0.063
Uric acid (mg/dL)	4.30 (2.50–6.20)	3.85 (2.70–6.80)	0.673	4.18 (1.01)	3.95 (0.87)	**0.027**	−0.06 (0.75)	−0.23 (0.52)	0.321

Values are presented as mean (standard deviation) or median (minimum–maximum value). The *p* values represent the results obtained by the Paired *t*‐test or Wilcoxon, and ∆*p* (final − initial) represents those obtained by the independent samples t‐test or Mann–Whitney U test. Values with statistically significant difference indicated in bold. µUI/mL: international micro unit per milliliter; Apo A: apolipoprotein A; Apo B: apolipoprotein B; TC: total cholesterol; HDL‐c: high‐density lipoprotein; HOMA‐IR: homeostasis model assessment—insulin resistance; HOMA‐β: homeostasis model assessment—beta cells; LDL‐c: low‐density lipoprotein; mg/dL: milligram per deciliter; TG: triglycerides; TyG; triglycerides/glucose index; VLDL‐c: very low‐density lipoprotein.

**FIGURE 3:**
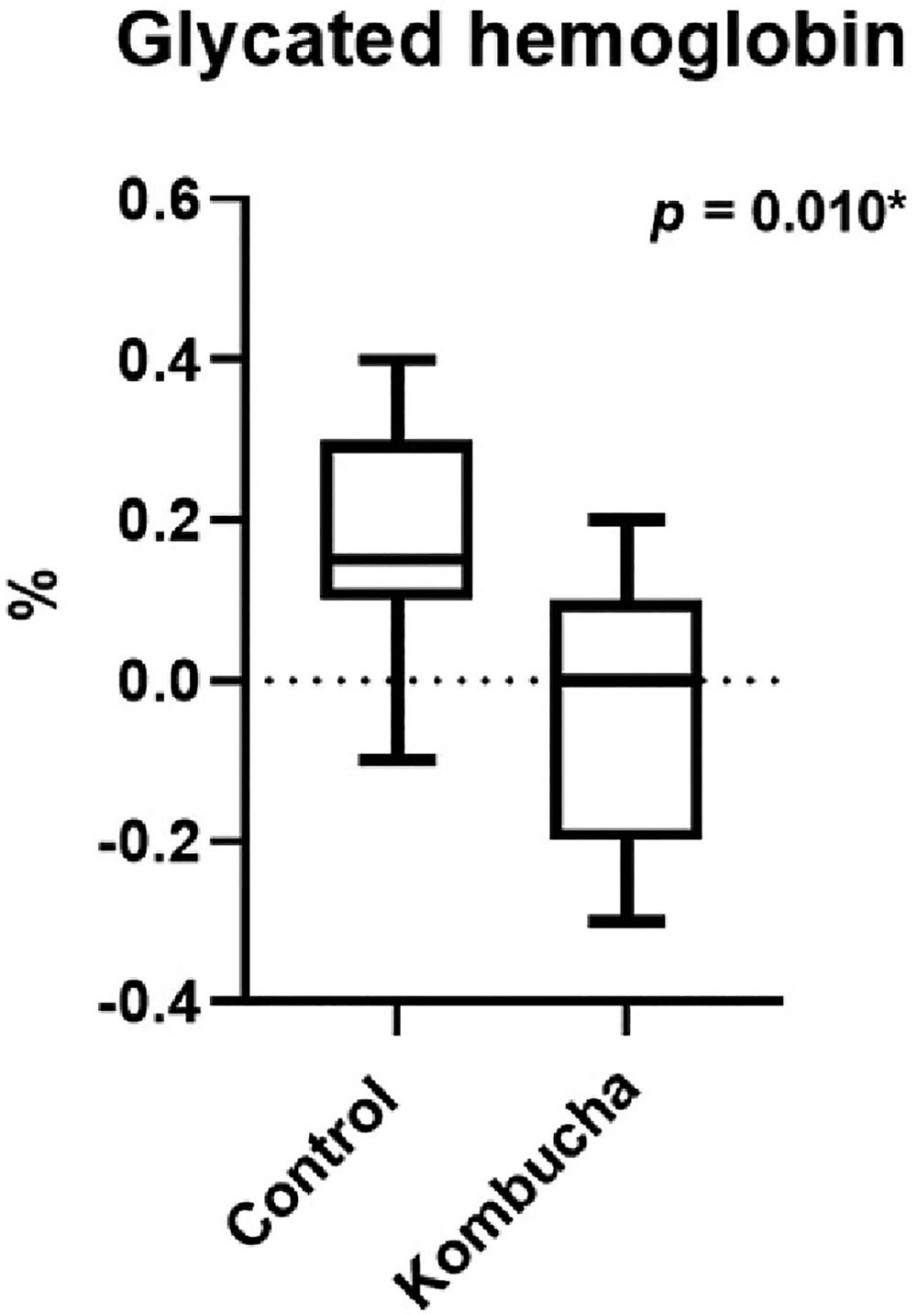
Effect of interventions on glycated hemoglobin in men participants of both groups. The *p* value represents the differences between the *∆* (final − initial) of the kombucha and control group in the independent samples *t*‐test. In the comparative analysis including all participants (men and women), no differences were observed between the groups.

Factorial ANOVA revealed a significant sex‐related difference in glycated hemoglobin (*p* = 0.049). Post‐hoc comparison tests between groups showed that men in the kombucha group reduced glycated hemoglobin, while men in the control group increased this marker (Figure 3).

## 4 Discussion

Although no significant differences were found in the comparison between the total groups at the two time points (*∆* = final − initial), the intragroup results indicate that daily consumption of 200 mL of green tea kombucha, in the context of an energy‐restricted healthy diet lasting 10 weeks, is a promising beverage for cardiometabolic benefits. The kombucha group significantly reduced lipid profile markers, such as TC, LDL‐c, VLDL‐c, TG, the Castelli II index, and uric acid compared to baseline levels. These improvements were not observed in the control group, which also did not exhibit any additional beneficial effect in relation to the kombucha group. Both groups achieved similar reductions in the Castelli I index and HOMA‐β (*p* < 0.05), likely attributable to the energy‐restricted diet. At the end of the study, only the control group increased glycated hemoglobin, though this remained within the normal range. Notably, among male participants, the kombucha intervention was more effective in reducing glycated hemoglobin compared to the control group, with a significant difference between the two.

The beneficial effect of green tea kombucha on the lipid profile, in the context of an energy‐restricted healthy diet, can be attributed to its rich composition, which includes 92 types of phenolic compounds, mostly from the flavonoid class (70.7%). Green tea itself is naturally abundant in complex polyphenols (Zhao et al. 2019), and during the fermentation process of kombucha, these compounds are broken down by enzymes released by bacteria and yeast. This degradation produces simpler molecules and increases the total phenolic content of the beverage (Bhattacharya et al. 2013). Notable compounds formed during the fermentation include gallocatechin, catechin 5‐O‐gallate, epicatechin, epigallocatechin, 5‐O‐galloylquinic acid, catechin, 5‐p‐coumaroylquinic acid, quercetin 3‐O‐rutinoside, and niricetin 3‐O‐glycoside. Additionally, acetic acid bacteria present in kombucha can convert glucose into gluconic acid (Villarreal‐Soto et al. 2018), which in turn conjugates with phenolic compounds, enhancing their bioavailability (Martínez Leal et al. 2018). This transformation further amplifies the functional properties of the beverage, contributing to its lipid‐lowering effects.

A review of the literature on kombucha highlights that phenolic compounds can stimulate sirtuin‐1 (SIRT‐1), activate adenosine monophosphate‐activated protein kinase (AMPK), and inhibit the transcription of sterol regulatory element‐binding protein 2 (SREBP). Collectively, these mechanisms contribute to metabolic homeostasis by reducing lipogenesis and cholesterol synthesis in the liver. Moreover, kombucha, through its phenolic compounds and probiotic microorganisms, can confer benefits to the intestinal microbiota, resulting in systemic effects such as improvements in the lipid profile (Costa et al. 2023). Furthermore, catechins, abundantly present in green tea kombucha, are known to inhibit lipid absorption and emulsification, further supporting its lipid‐lowering properties (Cardoso et al. 2021).

Consistent with the findings of the present study, the consumption of green tea kombucha for 16 weeks significantly reduced TC, TG, LDL‐c, and VLDL‐c levels in rats with hypercholesterolemia (Bellassoued et al. 2015). Similarly, another study involving rats fed a high‐fat, high‐fructose diet to induce metabolic alterations demonstrated that the consumption of green tea kombucha for 10 weeks reduced TG levels and reversed diet‐induced hepatic steatosis from grade 2 to grade 1. Furthermore, the same study revealed that kombucha activated pathways involving carnitine palmitoyltransferase I (CPT‐1), an enzyme crucial for fatty acid oxidation, which contributed to the reduction of triacylglycerols levels (Cardoso et al. 2021). Increased consumption of fructose and salt is well‐established as a contributing factor to obesity and metabolic syndrome, as these dietary components can activate purine nucleotide degradation pathways, leading to elevated uric acid levels (Andres‐Hernando et al. 2021). In this context, the effect of green tea kombucha, when combined with an energy‐restricted healthy diet, on reducing serum uric acid concentrations emerges as a significant cardiometabolic benefit. Supporting this, an in vitro study using proximal tubular cell line model cells (HK‐2) evaluated the effects of black tea kombucha and cascara coffee on oxidative stress. The study found that kombucha effectively reduced uric acid levels, highlighting its potential role in mitigating oxidative stress and promoting metabolic health (Sales et al. 2023).

Evidence suggests that the polyphenols in green tea can reduce uric acid levels by inhibiting the activity of xanthine oxidase, an enzyme that catalyzes purine reactions, transforming them into uric acid, and decreasing the expression of renal urate transporters, thereby enhancing uric acid excretion (Chen et al. 2015). Moreover, the literature identifies gallic acid as the primary bioactive compound in green tea with the potential to lower uric acid levels (Wu et al. 2022). The kombucha provided to participants contained a total phenolic content equivalent to 0.32 mg/mL of gallic acid, corresponding to a daily intake of 64 mg, based on the 200 mL portion offered.

Abdominal obesity is a known risk factor for cardiovascular diseases and insulin resistance (Piché et al. 2020). Additionally, glycated hemoglobin is considered the gold standard for measuring glucose levels, due to its high reliability and stability, reflecting average glucose concentrations over 2 to 3 months (Zhan et al. 2022). The observed reduction in glycated hemoglobin levels among men with excess body weight who consumed green tea kombucha is noteworthy. Men in this study demonstrated greater responsiveness to kombucha treatment, a difference that may be attributed to biological variations in glucose metabolism between men and women. Studies have shown that women tend to have lower glucose tolerance (Blaak 2008), reduced hepatic insulin extraction (Basu et al. 2006), and diminished fasting glucose uptake by the liver (Keramida and Peters 2017), which may have limited the significant effects of kombucha in this group.

Kombucha, through phenolic compounds, is capable of inhibiting glucose transporter 1 (GLUT1) and other enzymes of glucose metabolism, such as glycosity, in addition to inhibiting glucose absorption in the small intestine (Costa et al. 2023). Previous clinical studies have also identified the great hypoglycemic potential of kombucha. A randomized, double‐blind, crossover pilot clinical trial in a hospital system aimed to evaluate the effect of consuming 240 mL of kombucha on hyperglycemia in patients with type 2 diabetes *mellitus* for 4 weeks and identified that the beverage was able to reduce levels of blood glucose (Mendelson et al. 2023). Another clinical study also observed the postprandial hypoglycemic potential of kombucha in healthy people (Atkinson et al. 2023).

A healthy diet with calorie restriction is considered the main intervention to be adopted to reduce cardiometabolic risk markers in individuals with excess body weight who do not present comorbidities (Pepe et al. 2023), as observed in the participants in the present study. These effects were evidenced by the reduction of the Castelli I index and HOMA‐β in both groups. However, by the end of the study, participants in the control group exhibited a slight increase in glycated hemoglobin levels. This increase, less than 0.1%, did not surpass the normal threshold, defined as values below <5.7% (Little et al. 2019). This minor change is likely attributable to the body's adaptation to the caloric deficit, which initially leads to increased glycogenolysis (Most and Redman 2020) and, consequently, higher levels of free glucose available for hemoglobin glycation. It is plausible that the hypoglycemic properties of kombucha mitigated this effect in the group that consumed the beverage, further highlighting its potential role in managing glucose levels during dietary interventions.

One limitation of this study is the dosage of kombucha provided to participants, as there is currently a lack of studies and clinical guidelines that specify ideal doses for achieving therapeutic effects. To address this, the researchers opted for a safe supplementation dose, based on an approximate amount reported in a previous study (Mendelson et al. 2023). In addition, neither the researchers nor the participants were blinded to group allocation in this study. For future studies, certain aspects of the clinical design could be improved, such as a larger sample size, implementation of blinding, and a longer intervention duration, which may enhance the ability to detect the effects of kombucha on cardiometabolic risk markers.

Among the strengths of this study, the similarity between groups regarding participants' characteristics at baseline and the meticulous control of dietary intake throughout the study stand out. Finally, it is worth emphasizing that this is the first study dedicated to evaluating the effects of green tea kombucha consumption on cardiometabolic risk markers in individuals with excess weight.

## 5 Conclusion

The daily consumption of 200 mL of green tea kombucha for 10 weeks, combined with a healthy diet restricted by 500 kcal/day, reduced TC, LDL‐c, VLDL‐c, TG, Castelli II index, and uric acid in individuals with excess body weight (without apparent comorbidities) compared to baseline. Among men, green tea kombucha also led to a reduction in glycated hemoglobin levels when compared to the control group. These findings support the inclusion of green tea kombucha, alongside a caloric deficit, as part of nutritional strategies aimed at reducing cardiometabolic risk in individuals with excess weight. Given its rich composition of phenolic compounds, organic acids, and potentially beneficial microorganisms, further research is warranted to explore new therapeutic applications and targets for green tea kombucha.

## Author Contributions

**Dandara Baia Bonifácio**: conceptualization, methodology, formal analysis, investigation, writing – original draft, writing – review and editing, visualization. **Gabriela Macedo Fraiz**: methodology, investigation, writing – original draft, writing – review and editing, visualization. **Udielle Vermelho Lacerda**: methodology, investigation. **Rodrigo Rezende Cardoso**: methodology, investigation. **Thayná de Souza Coura Dias**: methodology, investigation. **Viviana Corich**: methodology, resources, project administration, funding acquisition. **Alessio Giacomini**: resources, methodology, project administration, funding acquisition. **Frederico Augusto Ribeiro de Barros**: conceptualization, methodology, resources, writing – original draft, writing – review and editing, project administration, funding acquisition, supervision. **Josefina Bressan**: conceptualization, methodology, resources, writing – original draft, writing – review and editing, supervision, project administration, funding acquisition.

## Funding

This study was funded by FAPEMIG (grant number: APQ‐00035‐20), and CAPES (code: 001).

## Conflicts of Interest

The authors declare no conflicts of interest.

## Data Availability

The remaining data from this article are available upon request.
